# Pre–Post Changes in Mood States Following a Single Hatha Yoga Session in Adult Women: A Community-Based Study

**DOI:** 10.3390/healthcare14091122

**Published:** 2026-04-22

**Authors:** Eleftheria Morela, Evgenia Kouli, Evangelos Galanis, Nerantzoula Koufou, Konstantinos Astrapellos

**Affiliations:** 1 Department of Physical Education and Sport Science, Democritus University of Thrace, University Campus, 69100 Komotini, Greece; ekouli@phyed.duth.gr (E.K.); nkoufou@phyed.duth.gr (N.K.); kastrape@phyed.duth.gr (K.A.); 2 Department of Physical Education and Sport Science, University of Thessaly, Karies, 42100 Trikala, Greece; egalanis@uth.gr

**Keywords:** Profile of Mood States (POMS), yoga practice, mental health, mind–body exercise, single session

## Abstract

**Background:** Hatha yoga has gained increasing popularity worldwide and has been associated with benefits for mental health and short-term emotional functioning. **Objective:** The present study examined pre–post changes in mood states following a single Hatha yoga session in adult women participating in community-based exercise programs. **Methods:** A total of 253 adult women participated in the study. Participants completed the Profile of Mood States (POMS) questionnaire immediately before and after a single 60 min Hatha yoga session. The questionnaire assesses anxiety–tension, depression, anger, fatigue, confusion, and vigor. Repeated measures ANOVA was used to examine the changes in mood states and the potential differences between the age groups. **Results:** Significant improvements in mood states were observed following the session. Anxiety–tension, depression, anger, fatigue, and confusion decreased, while vigor increased. No significant time × age group interaction was observed for most mood variables. However, a significant interaction was found for vigor, indicating that women aged 41 and older showed a greater increase following the session. **Conclusions:** Participation in a single Hatha yoga session was associated with short-term changes in mood states among adult women, suggesting that yoga may represent a potentially beneficial community-based activity for supporting short-term mood regulation.

## 1. Introduction

Rooted in ancient traditions, yoga integrates physical postures (asanas), breathing techniques (pranayama), relaxation, and meditation (dhyana) to foster both physical and mental well-being [1]. In recent decades, population-based surveys have documented a steady increase in yoga participation worldwide. In a nationally representative U.S. sample, Davis and colleagues [2] reported that approximately 16.8% of adults practiced yoga in 2022, reflecting a substantial growth over the past 20 years. In Europe, the percentage of people practicing yoga ranges from 12% to 20% in countries such as Poland [3], the UK [4], and Iceland [5]. In addition, during challenging times such as the COVID-19 pandemic, yoga appeared to play a critical role in managing stress and maintaining a sense of connection despite restrictions on physical interaction [6]. These patterns suggest that yoga has become an increasingly embedded component of contemporary health behavior and highlight the importance of examining potential benefits.

Extensive research has documented both the physical and mental health outcomes of yoga practice across various contexts [7]. Among the physical health benefits, yoga has been associated with improvements in flexibility, endurance, muscular strength, and cardiorespiratory fitness [8,9,10]. These benefits have been observed across diverse populations, including individuals engaging in yoga for general fitness [10], older adults [11], and various clinical populations [12,13], thus supporting its utility as an effective physical practice across a range of health contexts. This evidence across different health conditions suggests that yoga is a highly adaptable practice with potential relevance for both preventive health strategies and clinical applications.

Yoga has also been associated with positive psychological outcomes across diverse populations, from school-aged children [14] to older adults [15]. Systematic reviews and meta-analyses have confirmed that yoga-based interventions are effective in reducing symptoms of stress, anxiety, depression, and burnout, and have also been shown to enhance well-being, resilience, and emotional functioning across a wide range of populations [16,17,18]. More recent intervention-focused reviews further confirm these findings in mentally demanding contexts, reporting reductions in stress, anxiety, burnout, and depressive symptoms among healthcare professionals and students participating in yoga programs [19,20]. Similarly, findings from cross-sectional studies suggest that individuals who engage in regular yoga practice report higher levels of psychological well-being compared to non-practitioners [21], while qualitative research highlights yoga’s perceived role in supporting long-term mental health and sustained self-care practices [22]. Overall, the literature indicates that yoga practice is associated with reduced negative effects and enhanced adaptive emotional functioning.

Recent research has begun to explore the underlying mechanisms of mind–body interventions. For example, Han and colleagues [23] demonstrated that practices such as yoga, tai chi, and qigong are associated with changes in brain regions involved in attention, self-awareness, and emotional regulation, including networks linked to stress reduction and mood improvement. Similarly, Remskar and colleagues [24] reported that interventions combining physical activity with mindfulness are associated with greater improvements in stress and anxiety, potentially through affective mechanisms such as enhanced emotion regulation, increased interoceptive awareness, and reduced rumination. These findings suggest that mind–body practices may influence psychological outcomes by simultaneously targeting physiological and cognitive–emotional processes.

Among the documented mental health benefits of yoga practice, long-term participation has been linked with improved mood-related outcomes, including lower levels of anxiety, anger, and fatigue [25,26]. Emerging evidence also suggests that such benefits are not limited to sustained practice. A growing body of research indicates that even a single yoga session can produce measurable short-term psychological effects. For instance, studies have reported reductions in anxiety and overall mood disturbance following brief yoga sessions in both non-clinical and clinical populations [27,28,29]. Supporting these findings, a recent systematic review found that a substantial proportion of studies examining acute responses to yoga reported reductions in psychological stress outcomes, such as perceived stress and anxiety [30]. In addition, Tartar and colleagues [31] demonstrated that even a single session of yoga practice can lead to immediate improvements in mood states, including increased vigor and reductions in fatigue and confusion.

Despite growing evidence on both the long-term and short-term psychological benefits of yoga, relatively little is known about the immediate effects of a single yoga session on broader mood states in everyday community settings. As mood states, including overall mood disturbance, are sensitive indicators of acute psychological responses to physical activity, examining changes in mood before and after a single yoga session in everyday settings appears particularly relevant. It is therefore important to distinguish between mood states and broader psychological well-being. Mood states are generally defined as transient, situation-dependent affective responses that fluctuate over short periods of time [32], whereas psychological well-being represents a more stable and multidimensional evaluation of positive functioning and life experiences [33].

The purpose of the present study was to examine pre–post changes in mood states following participation in a single yoga session, including the overall mood disturbance, among adult women participating in community exercise programs organized by the municipality. It was hypothesized that participation in a single yoga session would be associated with improvements in mood, reflected by lower levels of negative mood indicators and reductions in overall mood disturbance, along with increases in positive mood indicators. In addition, building on evidence suggesting that yoga may influence psychological functioning across different adult populations [34], the present study further explored potential age-related variations in mood outcomes. To address these aims, the study was conducted as an observational pre–post field investigation in a real-world community setting without a control group; thus, the findings reflect short-term associations rather than causal effects.

## 2. Materials and Methods

### 2.1. Procedures and Participants

The present study employed a one-group pre–post observational design conducted in a naturalistic community setting. As no control or comparison group was included, the design does not permit causal inferences. The study was conducted in accordance with the guidelines of the Declaration of Helsinki and was approved by the Institution’s Ethics Committee. The municipality-organized community exercise programs were selected because they represent accessible, community-based initiatives that promote regular physical activity among adult women across different age groups. These programs were conducted during the afternoon hours in local public-school facilities, as part of the municipality’s broader community exercise initiatives. The city of Komotini was initially chosen due to the researchers’ accessibility and collaboration with the local municipality. Before data collection, all municipality-organized yoga programs in Komotini were identified, and the program coordinators were contacted by the researchers, who explained the purpose of the study and asked them to participate. After approval, the researchers provided the yoga instructors with the study’s questionnaires and detailed instructions regarding their administration.

The study adhered to the ethical principles of voluntary participation, anonymity, and confidentiality. Before the data collection, participants were informed about the purpose of the research and provided written informed consent. They were assured that participation was voluntary and that they could withdraw from the study at any time. Data collection took place during regularly scheduled yoga sessions that were part of municipality-organized community exercise programs held in local public-school facilities. All participants attended a single 60 min Hatha yoga session conducted within the framework of the municipality-organized exercise programs. Sessions followed a general structure consisting of a brief warm-up phase (approximately 10 min), a sequence of yoga postures (asanas) with integrated breathing techniques (approximately 40 min), and a final relaxation phase (approximately 10 min), in line with established Hatha yoga models described in the literature [35]. The sessions emphasized controlled breathing, body awareness, and gradual transitions between postures, consistent with commonly applied Hatha yoga practices. The sessions were delivered by certified instructors involved in the municipal exercise programs. As multiple instructors were involved across different programs, a fully standardized session protocol was not implemented. However, all sessions followed the general structure of the municipal yoga curriculum (i.e., warm-up, sequence of postures with breathing integration, and relaxation), which provided a degree of consistency in the session delivery, although some variation in instruction style and emphasis may have occurred. Participants completed the questionnaires immediately before the session began and shortly after its completion.

A total of 253 adult women aged 21 to 59 years participated in the study. Participants were recruited from municipality-organized community exercise programs offering yoga programs in Komotini, Greece. Participation was based on voluntary enrollment in the municipality-organized yoga programs, and no additional inclusion or exclusion criteria were applied beyond attendance in these sessions. As such, the sample may reflect individuals who are already positively oriented toward physical activity and yoga participation. For the purpose of the analysis, participants were categorized into three age groups: 21–30 years (n = 128), 31–40 years (n = 56), and 41 and above (n = 69). This categorization was used to allow comparison across broad adult age ranges while maintaining sufficient subgroup sizes for statistical analysis; however, it may have limited the detection of more fine-grained age-related differences. The 41 and above category was treated as a single group because further subdivision would have resulted in smaller cell sizes and reduced analytical stability. However, this broader age grouping may have obscured heterogeneity within older participants. No additional information was systematically collected regarding participants’ prior yoga experience, mental health status, or detailed physical activity level, and these factors were not controlled in the present study. Consequently, the representativeness of the sample and the interpretation of the pre–post responses should be considered within the context of the limited baseline participant characterization.

### 2.2. Instrument

Participants completed the short form of the Profile of Mood States (POMS) questionnaire [36], which assesses momentary emotional state and general mood dispositions. The questionnaire consists of 37 items (short form) introduced by the prompt “How are you feeling right now?” and they are grouped into six subscales: (a) anxiety–tension (6 items; e.g., anxious), (b) depression (8 items; e.g., sad), (c) anger (7 items; e.g., irritated), (d) vigor (6 items; e.g., full of energy), (e) fatigue (5 items; e.g., exhausted), and (f) confusion (5 items; e.g., confused). Items are rated on a five-point Likert scale ranging from 0 (not at all) to 4 (extremely). Subscale scores were calculated as the mean of the corresponding items. The “right now” response format makes it possible to assess short-term mood changes occurring immediately before and after exercise, thereby focusing on the acute psychological effects of a single exercise session. It should be noted that the subscales of the POMS (e.g., anxiety and depression) refer to transient, self-reported mood states and do not correspond to clinical diagnostic frameworks such as the DSM-5. Therefore, the present scale does not assess clinical conditions but rather short-term fluctuations in affective states. High vigor scores and low scores on the remaining subscales indicate a more positive mood state. The total mood disturbance (TMD) score was computed by summing the five negative subscale scores (anxiety–tension, depression, anger, fatigue, and confusion) and subtracting the vigor score, with the addition of a constant of 100, in accordance with standard POMS scoring procedures. As TMD represents a composite index derived from multiple subscales rather than a distinct latent construct, internal consistency (Cronbach’s alpha) was not calculated for this score. Based on the scoring procedure used (including the addition of a constant), TMD scores are centered around 100, with higher values indicating greater overall mood disturbance. The POMS short form has demonstrated satisfactory factorial structure, reliability, and construct validity in previous research [36]. The Greek adaptation [37] has shown adequate internal consistency in subsequent studies, including recent research with older adults in Greece [38].

### 2.3. Data Analysis

The internal consistency of the questionnaire was assessed using Cronbach’s alpha coefficients. Descriptive statistics (means and standard deviations) were calculated for all mood variables before and after the yoga session. Mood changes were examined using repeated measures analyses of variance (ANOVA), with time (pre, post) as the within-subjects factor and age group (21–30, 31–40, and 41+) as the between-subjects factor. Effect sizes are reported as partial eta squared (*η*^2^). Because the within-subjects factor included only two levels, the assumption of sphericity was not applicable. The assumptions of normality were examined through inspection of distributions, and no substantial deviations were observed. Missing data were handled using listwise exclusion within each analysis. Given the number of statistical tests performed, no formal correction for multiple comparisons was applied, and the results should be interpreted with caution. Values of *p* < 0.05 were considered statistically significant.

## 3. Results

Data analyses confirmed internal consistency. Cronbach’s alpha coefficients for all factors ranged from 0.65 to 0.93 before participation, and from 0.53 to 0.91 after participation. The means (M), standard deviations (SD), and Cronbach’s alpha coefficients for the questionnaire factors and total mood disturbance (TMD) before and after yoga practice are presented in Table 1.

Repeated measures analyses were conducted to examine changes in participants’ mood states following participation in the yoga session. Specifically, repeated measures analyses of variance were used to assess pre–post changes in the total mood disturbance, as well as in the individual mood factors (anxiety–tension, depression, anger, vigor, fatigue, and confusion), and to explore the potential differences related to age group.

### 3.1. Total Mood Disturbance (TMD)

Regarding total mood disturbance (TMD), repeated measures ANOVA revealed a significant main effect of time, F(1, 246) = 347.96, *p* < 0.001, *η*^2^ = 0.59, indicating a decrease in TMD following the yoga session. The time × age group interaction was not statistically significant, F(2, 246) = 0.60, *p* = 0.55, *η*^2^ = 0.005, indicating that the magnitude of improvement was similar across age groups. The main effect of age group was also not significant. However, exploratory pairwise comparisons indicated that women aged 21–30 years reported higher TMD scores than women aged 41 and older (*p* < 0.05). Means (M) and standard deviations (SD) for the total mood disturbance are presented in Table 2.

### 3.2. Mood Factors

Repeated measures ANOVA revealed a significant main effect of time for all mood factors. Anxiety–tension decreased, F(1, 250) = 218.18, *p* < 0.001, *η*^2^ = 0.46; depression decreased, F(1, 248) = 165.88, *p* < 0.001, *η*^2^ = 0.40; anger decreased, F(1, 250) = 128.23, *p* < 0.001, *η*^2^ = 0.34; fatigue decreased, F(1, 248) = 11.05, *p* < 0.001, *η*^2^ = 0.04; and confusion decreased, F(1, 249) = 167.09, *p* < 0.001, *η*^2^ = 0.40. In contrast, vigor increased significantly following the yoga session, F(1, 249) = 255.46, *p* < 0.001, *η*^2^ = 0.51.

Moreover, no significant time × age group interactions were observed for anxiety–tension (*p* = 0.31), depression (*p* = 0.97), anger (*p* = 0.90), fatigue (*p* = 0.59), or confusion (*p* = 0.22), indicating that improvements in these mood states were comparable across age groups. The significant main effects of age group were observed for anxiety–tension, F(2, 250) = 3.67, *p* < 0.05, *η*^2^ = 0.03, and confusion, F(2, 249) = 5.55, *p* < 0.05, *η*^2^ = 0.04, with women aged 21–30 years reporting higher scores than women aged 41 and older. A significant time × age group interaction was observed for vigor, F(2, 249) = 7.70, *p* = 0.001, *η*^2^ = 0.05. Post hoc comparisons indicated that vigor increased significantly from pre- to post-session in all age groups, with the largest increase observed among women aged 41 and older. Significant differences were observed between the 21–30 and 41+ age groups. Means (M) and standard deviations (SD) for the mood factors before and after the yoga practice are presented in Table 2.

## 4. Discussion

The potential of yoga practice to enhance psychological well-being and positively influence mood has received considerable attention in both clinical and non-clinical populations [17,18]. Therefore, the present study examined the effects of a single yoga session on mood states among adult women participating in municipality-organized exercise programs and additionally explored potential age differences in mood outcomes. The findings revealed significant improvements in mood states following the yoga session, characterized by reductions in negative mood dimensions—namely anxiety–tension, depression, anger, fatigue, and confusion—and increases in vigor, thus supporting the research hypotheses regarding short-term mood changes. Notably, the magnitude of the observed effects suggests that these changes were not only statistically significant but also potentially meaningful within the context of short-term mood responses. These results are consistent with previous research indicating that even a single yoga session can reduce negative mood states [27,31] and enhance vigor [31] among diverse populations. In this respect, the potential value of a single yoga session is suggested particularly for individuals who may face barriers to sustained participation, such as limited time or restricted access to long-term exercise programs. However, it should be noted that the present findings relate specifically to short-term changes in self-reported, subclinical mood states and do not directly assess broader psychological well-being or clinically diagnosed conditions. Therefore, the results should be interpreted within the context of acute affective responses rather than long-term psychological outcomes. In addition, the absence of a control or comparison group limits causal interpretation, and the findings should therefore be understood as reflecting associations rather than definitive effects of the yoga session. In this context, alternative explanations for the observed changes should also be considered. These may include expectancy effects, social interaction within the group setting, measurement reactivity associated with repeated assessment, time-of-day influences, or general post-exercise mood enhancement, rather than the specific effects of yoga practice itself. Furthermore, the involvement of multiple instructors across programs may have introduced variability in session delivery, which could have influenced the participants’ responses.

In addition to examining the overall effects of a single yoga session on mood, the present study sought to further explore whether these effects varied across age groups. Overall, improvements in mood states following the yoga session were largely comparable across age groups, as no significant interaction was observed between the time and age group for negative mood states. This finding implies that women of different ages experienced similar improvements in anxiety–tension, fatigue, and confusion following yoga practice. However, significant main effects of age group were observed for anxiety–tension, fatigue, and confusion, with younger women (21–30 years) reporting generally higher levels than women aged 41 and above. These findings resemble those reported by Fields and colleagues [39], who found that younger adults tend to experience higher levels of stress and negative affect, whereas older adults often report better emotional regulation and lower distress. Despite these comparable improvements, a closer examination of age-specific patterns may shed light on differential responses across specific mood dimensions.

In contrast, a different pattern was observed for vigor, with a significant interaction between the time and age group, indicating that women aged 41 and above experienced a greater increase following the yoga session. One possible interpretation of this finding, which should be considered exploratory, is that it may be understood in light of socioemotional selectivity theory, which suggests that as people age, they place greater emphasis on regulating emotions and maximizing positive in-the-moment experiences [40]. From this perspective, older women may be more receptive to the immediate energizing and uplifting effects of yoga, which may help explain the greater short-term increase in vigor observed in this age group. These findings appear to reflect differences in responsiveness to the yoga session rather than stable age-related differences in vigor, as no significant differences between the age groups were evident at either measurement point. This interpretation should therefore be viewed as hypothesis-generating and requires further investigation.

With respect to overall mood, participation in a single yoga session was associated with a significant reduction in total mood disturbance across all age groups, suggesting a general improvement in psychological mood after the yoga session. Age-related differences were also evident in the pre-session measure, as women aged 21–30 years reported higher overall levels of mood disturbance compared to those aged 41 and above. This finding aligns with previous evidence indicating higher psychological distress and negative affect among young adults [41]. Overall, the findings of the present study contribute to the still-limited literature examining the acute psychological effects of yoga participation in community-based settings. In line with previous research demonstrating that even a single yoga session can lead to improvements in mood-related outcomes [29,42], the present study suggests that yoga may represent a potentially useful and accessible form of physical activity for supporting short-term mood regulation among adult women in publicly available exercise programs.

## 5. Limitations and Future Research Directions

Despite these encouraging findings, several limitations should be acknowledged. First, the absence of a control or comparison group limits the internal validity of the study and precludes causal interpretation of the findings. As such, the observed changes in mood states may be influenced by non-specific factors, including participant expectations, social interaction, measurement reactivity, time-of-day effects, or general engagement in physical activity rather than the specific effects of yoga practice. In addition, the involvement of multiple instructors across the programs may have introduced variability in session delivery, which could have influenced the participants’ responses. Second, the use of a single yoga session restricts the extent to which the results can be generalized to longer-term yoga practice and does not allow conclusions about the durability of mood changes over time to be drawn. Furthermore, participants were recruited from municipality-organized exercise programs and were already engaged in structured physical activity, which may introduce a selection bias and limit the generalizability of the findings to less active populations. Third, limited participant characterization represents an additional constraint. Detailed information regarding prior yoga experience, overall physical activity levels, or mental health status was not systematically collected, which limits the ability to fully interpret individual differences in responses to the yoga session. The use of a broader age category for participants aged 41 and above may have further obscured potential heterogeneity within this subgroup. These limitations also constrain the interpretation of the observed changes, as individual differences related to prior experience or baseline characteristics could not be accounted for. It is also possible that the participants’ prior interest in or positive attitudes toward yoga influenced their responses, potentially contributing to expectancy effects.

Finally, mood was assessed using self-report measures administered immediately before and after the session. Although appropriate for capturing short-term changes, such measures may be influenced by response bias or situational factors. In addition, the internal consistency of some post-test subscales was relatively low, possibly due to reduced variability in responses and floor effects following the session, as well as the acute nature of the assessment. Additional analytical limitations should also be considered. No formal correction for multiple comparisons was applied, and confidence intervals for mean changes were not reported, which may affect the interpretation and precision of the observed effects. Therefore, these findings should be interpreted with caution.

In light of these limitations, several directions for future research can be identified. Future research should further examine the persistence of these acute mood changes through longitudinal or repeated-session designs, incorporate appropriate comparison conditions, and include more diverse samples. In addition, integrating objective or physiological indicators may help to clarify the mechanisms underlying mood-related responses to yoga practice. Where ethically feasible, future work should also consider facilitating reproducibility through the use of anonymized data sharing practices.

## 6. Conclusions

Τhe present study suggests that participation in a single Hatha yoga session was associated with short-term improvements in mood among adult women attending municipal exercise programs. Consistent with previous findings demonstrating the acute mood-enhancing effects of yoga across diverse populations [27,31], the results indicate reductions in negative mood states alongside increases in vigor. By situating the investigation within publicly accessible, community-based exercise settings, this study points to a potential role for yoga as a feasible approach to supporting short-term improvements in mood regulation in everyday contexts. Overall, the findings contribute to the emerging literature on acute mood responses to yoga in real-world community settings.

## Figures and Tables

**Table 1:** Means, standard deviations, and Cronbach’s alpha coefficients for the questionnaire factors and total mood disturbance (TMD) before and after yoga practice.

Measurements						
Factors	Pre			Post		
	M	SD	α	M	SD	α
Anxiety–Tension	0.91	0.70	0.74	0.23	0.32	0.53
Depression	0.59	0.62	0.82	0.17	0.30	0.73
Anger	0.46	0.56	0.76	0.12	0.28	0.71
Vigor	1.55	1.03	0.93	2.72	0.96	0.91
Fatigue	0.74	0.73	0.84	0.57	0.65	0.74
Confusion	0.87	0.67	0.65	0.39	0.47	0.61
TMD	102.01	2.96		98.75	1.97	

**Table 2:** Means and standard deviations for the questionnaire and total mood disturbance (TMD) before and after the yoga practice.

Factor	21–30 Years		31–40 Years		41+ Years	
	Pre (M ± SD)	Post (M ± SD)	Pre (M ± SD)	Post (M ± SD)	Pre (M ± SD)	Post (M ± SD)
Anxiety–Tension	1.00 ± 0.73 *	0.27 ± 0.35	0.87 ± 0.72	0.26 ± 0.30	0.76 ± 0.62 *	0.15 ± 0.25
Depression	0.63 ± 0.68	0.22 ± 0.33	0.53 ± 0.58	0.10 ± 0.28	0.56 ± 0.52	0.13 ± 0.22
Anger	0.51 ± 0.62	0.17 ± 0.36	0.40 ± 0.54	0.07 ± 0.18	0.42 ± 0.45	0.06 ± 0.11
Vigor	1.70 ± 0.99	2.56 ± 0.98	1.43 ± 0.86	2.77 ± 0.75	1.35 ± 1.20	2.90 ± 1.04
Fatigue	0.79 ± 0.72 *	0.60 ± 0.69	0.85 ± 0.87	0.62 ± 0.56	0.57 ± 0.60 *	0.48 ± 0.63
Confusion	0.99 ± 0.68 *	0.47 ± 0.50	0.82 ± 0.73	0.33 ± 0.49	0.68 ± 0.55 *	0.29 ± 0.34
TMD	102.19 ± 3.19 *	99.11 ± 2.30	102.03 ± 2.97	98.61 ± 1.59	101.66 ± 2.46 *	98.19 ± 1.35

Notes: The values are presented as mean ± SD. * indicates a statistically significant difference between the 21–30 and 41+ age groups, *p* < 0.05.

## Data Availability

The data that support the findings of this study are available from the first author upon reasonable request. Due to privacy and confidentiality considerations related to the study participants, the datasets are not publicly available.

## Abbreviations

The following abbreviations are used in this manuscript:

TMD	Total Mood Disturbance
POMS	Profile of Mood States
